# A Community Coalition Board Creates a Set of Values for Community-based Research

**Published:** 2005-12-15

**Authors:** Daniel S Blumenthal

**Affiliations:** Department of Community Health and Preventive Medicine, Morehouse School of Medicine

## Abstract

**Background:**

Researchers generally agree that communities should participate in the community-based research process, but neither a universally accepted approach to community participation nor a set of guiding principles exists.

**Context:**

The Morehouse School of Medicine Prevention Research Center was established in 1999 with the support of a grant from the Centers for Disease Control and Prevention. Its partners include a low-income, predominantly African American community, six public agencies, and two other academic institutions. A Community Coalition Board was established to represent the partners. The majority of the board is community members; it serves in a governance rather than an advisory capacity, with the community acting as the senior partner in interactions with the medical school, the agencies, and other academic institutions.

**Methods:**

The Community Coalition Board developed a set of research priorities and a set of 10 community values, or principles, to guide research. A board committee reviews each protocol to ensure they uphold the values.

**Consequences:**

The Community Coalition Board has been using the values since 1999, and in this article we describe its experience. After an initial period that included some disagreements between researchers and community members on the board, relationships have been good, and protocols have been approved with only minor changes.

**Interpretation:**

Although the established community values reflect universally acknowledged principles of research ethics, they also address local concerns. An equal partnership between community members and researchers is most beneficial if the partners can agree on a set of values to govern research.

## Background

During the last 30 years, researchers have been rethinking the relationship between researchers and research participants. *The* *Belmont Report* (1) outlined three ethical principles to help researchers protect the rights of research participants: respect for individuals (autonomy), beneficence, and justice. Subsequent reports have expanded and refined these principles (2,3). Simultaneously, community-based research has emerged, so researchers have had to consider the rights of communities participating in research in addition to the rights of individual participants (4-6).

Many approaches to community interactions with institutions and professionals have incorporated components of efforts during the 1970s to develop "maximum feasible community participation" (7-9). In 1971, Arnstein created a *ladder of citizen participation* that defined eight levels of participation in service projects (10). More than 20 years later, Hatch et al defined four levels of community participation in research projects (11). At Hatch's first level, researchers consult with people who work for human service agencies but usually do not live in the community. At the second level, community leaders are recruited to be project advisors, but the researchers retain control of the projects. At the third level, community leaders are asked to endorse the projects and assist with hiring community residents to serve in roles such as interviewers and outreach workers. At the fourth level, community representatives are *first among equals,* or senior partners. They define the research agenda, identify and analyze the problem to be studied, and propose possible solutions. In Hatch's model, the status of community representatives as first among equals differs from the community control model of the 1970s, in which the governance structure could have consisted entirely of community representatives.

Building on this background, several writers have defined ethical principles for community participation in research (12,13). Community-based research is now widely referred to as *community-based participatory research,* reflecting the general acknowledgement that community participation is desirable (14). However, the protection of research participants, whether individuals or communities, has traditionally been a topic addressed by researchers and ethicists, not by communities. We report one case study in which community participants created their own set of principles, or *community* *values,* to guide research being conducted in their community.

## Context

The Morehouse School of Medicine Prevention Research Center (PRC) was established in 1999 in Atlanta, Ga, with the support of a grant from the Centers for Disease Control and Prevention. The PRC was created in partnership with a low-income, predominantly African American community known as *Neighborhood Planning Unit Y (NPU-Y)*, which is one of 24 NPUs into which the city of Atlanta is divided. NPU-Y has a population of about 25,000 and comprises eight well-defined neighborhoods, each with its own neighborhood organization, and a publicly owned apartment building for senior citizens. Approximately 90% of the community residents are African American, and the median yearly household income is about $17,000. NPU-Y 1) holds a monthly meeting that can be attended by any resident, 2) elects officers, and 3) is recognized by the city (as are the other NPUs) as the advisory body on community matters.

When establishing an entity to represent the community during its interactions with the PRC, we avoided using the term *advisory* (e.g., Community Advisory Board) because of its implied powerlessness. Instead, we hoped to create a governance model in which the community would serve as the senior partner in its relationship with the medical school and other academic and agency collaborators. We therefore established a Community Coalition Board to which all the partners belong but on which community representatives hold the preponderance of power. Seats on the board were assigned to the medical school, two other academic institutions, six agencies (the health department, the public schools, the public housing authority, the local community health center, the area health education center, and the Empowerment Zone Corporation), and each of the neighborhoods in NPU-Y, as well as some adjoining neighborhoods. (The Atlanta Empowerment Zone was a federally designated inner-city area represented by a nonprofit corporation, the Empowerment Zone Corporation [now defunct], with the authority to award grants and offer tax concessions.) The bylaws of the Community Coalition Board state that community representatives must always hold the majority of the positions, and a community representative must serve as the chairperson.

The bylaws permit a maximum of 25 board members. At the beginning of fiscal year 2005 (October), 17 board positions had been filled — nine with community representatives, three with academic representatives, and five with agency representatives. The board chairperson was a retired elementary school teacher; the community membership was diverse and included a minister, an acupuncturist, a community center director, a television repairman, a computer analyst, a computer technician, an office worker, and a building contractor. Five of the nine community representatives were charter members of the board from 1998. None of the board members had served fewer than 4 years. (The bylaws do not specify a term of office.) Eight of the nine community members were African American.

## Methods

The Community Coalition Board established a research agenda for the PRC as well as a set of criteria against which it could review all research protocols. The board did not want to develop a disease-specific research agenda and therefore created a broad agenda called *Research Priorities* that expressed the board's concern with the overall poor state of health among African Americans, particularly African American males (Table 1).

The criteria established by the board for evaluating projects includes the statement, "They [the projects] should not violate community values or standards." One of the board members questioned exactly which community values were being considered. The result of this inquiry was a 6-month board effort to create the *Statement of Community Values* (Table 2). Some of the value statements that were created were modified from statements of other organizations, such as the National Association of Black Social Workers; others were generated to address board members' specific concerns. After each monthly meeting, the principal investigator revised each emerging statement, and the draft was presented at the following meeting for additional assessment and refinement. The process was repeated until a final statement was created.

## Consequences

The PRC's experience applying the values during its first 6 years of operation can be characterized by consideration of each value statement.

1. **Policies and programs should be based on mutual respect and justice for all people, free from any form of discrimination or bias.**
  Having programs and policies based on mutual respect is the fundamental ground rule that governs all PRC research. It specifically addresses the exploitative and discriminatory experiences that some minority populations have had with research and health care in the past. When the PRC was being organized, some community members openly expressed their lack of trust in the researchers. For instance, during one NPU-Y meeting, the idea of applying for a PRC grant was being presented. A community resident stood and said, "I grew up in Tuskegee, Alabama, and I know what you researchers are about. You will exploit the community for your own purposes." It is unlikely that our reassurances convinced her of our benign intent. Likewise, a community leader who later became a member of the Community Coalition Board told a group of faculty members: "I've seen university faculty in action. You want to gather some data, then go back to your offices and write your papers. I don't want to stand in your way, but don't expect me to help you." It was not immediately clear to the community that our research would be conducted on the basis of mutual respect and justice for all people; trust that we would adhere to this principle had to be developed over time. 
2. **All people have a right to political, economic, cultural, and environmental self-determination.**
  A conflict related to the value of self-determination arose over the initial location of the PRC offices. Community members of the board insisted that the PRC should be geographically located within NPU-Y. The other board members shared the view of the staff, which was that NPU-Y did not have a suitable office building. A compromise location was eventually found just outside the NPU-Y boundary, on the other side of an expressway. The principle of self-determination dictated that the community should decide the site of its research center, but the availability of a suitable building was a limiting factor.
3. **The community has the right to participate as an equal partner at every level of decision making, including needs assessment, planning, implementation, enforcement, and evaluation.**
  Serving as an equal partner means that the community members should not be overruled by the researchers. As previously mentioned, the bylaws established the community as first among equals by declaring that community representatives should hold the majority of seats and that one should serve as the chairperson of the Community Coalition Board. However, resource limitations have restricted the community's ability to make certain decisions. For instance, many of the board's community members were disappointed that the PRC did not have researchers with the expertise to pursue grants in some of their areas of interest, such as complementary and alternative medicine.
4. **Principles of individual and community informed consent should be strictly enforced.**
  Obtaining individual informed consent has not been an issue for the board, which has deferred to the Morehouse School of Medicine Institutional Review Board (IRB). However, the board has acted on behalf of the community in providing *community informed consent,* which is consent of the community, through appropriate representatives, to serve collectively as the subject of a research project. The consent process has been managed by a board committee consisting entirely of community members. With the assistance of a trusted professional, the committee reviews each research protocol. The committee has commented on every protocol and has recommended changes for most of them. The response of researchers to the recommended changes has usually been to alter the protocol to address community concerns, although one researcher walked out of a meeting and withdrew her proposal after hearing the recommended changes. Since the PRC's first year, criticisms have been relatively minor and have primarily consisted of requests that the projects focus more exclusively on NPU-Y.
5. **The community repudiates the targeting of people of color and lower socioeconomic status for the purpose of testing reproductive and medical procedures and vaccinations.**
  A perception exists that, historically, dangerous procedures, vaccines, and contraceptives have been tested on minority populations without adequate informed consent. None of the PRC projects has proposed to test reproductive or medical procedures or vaccinations.
6. **Present and future generations should be provided an education that emphasizes social and environmental issues, based on our experience and an appreciation of our diverse cultural perspectives.**
  The education principle is relevant for public schools and health promotion research. All PRC intervention projects have attempted to design culturally sensitive interventions.
7. **Research processes and outcomes should benefit the community. Community members should be hired and trained whenever possible and appropriate, and the research should help build and enhance community assets.**
  The community's most immediate needs are for services and jobs, not research, a principle that is emphasized in the PRC's *Research Priorities* (Table 1)*,* which states: "They [projects] should have the potential to benefit the community through a health promotion intervention." The PRC requires that all observational study proposals include an explanation of how they may be beneficial for the community. Of the first 14 projects conducted by the PRC, four were observational, eight consisted primarily of intervention testing, and two included both components. The PRC has also responded by hiring a community member as a core staff member and six community members as health workers on specific projects.
8. **Community members should be part of the analysis and interpretation of data and should have input into how the results are distributed. This does not imply censorship of data or of publication, but rather the opportunity to make clear the community's views about the interpretation prior to final publication.**
  A perception exists that researchers have a tendency to describe low-income minority communities in excessively negative terms, so we attempted to ensure that community members would have the opportunity to provide input on publications. In practice, community members of the board have expressed little interest in reviewing manuscripts before their submission to professional journals. However, this article was reviewed and approved by an ad hoc committee of the board, which consisted of three community members.
9. **Productive partnerships between researchers and community members should be encouraged to last beyond the life of the project. This will make it more likely that research findings will be incorporated into ongoing community programs and therefore provide the greatest possible benefit to the community from research.**
  The partnership between researchers and the community has indeed outlived individual projects, some of which have ended. The value statement affirms that the partnership itself is important and emphasizes that much of its significance is a direct result of the services provided by the partnership. Unfortunately, the services provided by expired projects often ended with the projects.
10. **Community members should be empowered to initiate their own research projects that address needs they identify themselves.**
  Through a program of minigrants, we conducted several workshops on writing proposals and then provided grants of up to $5000 to community organizations located in NPU-Y and adjacent communities. Five projects have been funded, and all have been service projects rather than research projects.

## Interpretation

During the deliberations of the Community Coalition Board that produced the *Research Priorities *and *Statement of* *Community Values,* the three principles in *The* *Belmont Report* (1) were never mentioned. Nonetheless, the 10 values clearly reflect the principles of beneficence (values 6, 7, 9, and 10), autonomy (values 2, 3, 4, and 8), and justice (values 1 and 5).

Thus, although the 10 values determined by the board represent universal principles of ethics, they are also tailored to represent the needs of the NPU-Y community. For instance, NPU-Y has many environmental issues. It surrounds an industrial plant that is thought by the community to be a source of air pollution. It is home to a sewage treatment plant, and several polluted creeks flow through the area. Anxiety is mounting about the potential loss of green space to developers. The environmental concerns are reflected in values 2 and 6.

The *Statement of Community Values* also reflects concerns that are typical of low-income communities but are often of less interest to researchers. The need for jobs is recognized in value 7. The overall need for education — not just on matters related to research projects — is reflected in value 6, whereas political and economic issues are addressed in value 2. Values 7 to 10 all suggest that the research program should benefit the local community; the overall benefits to humankind are not the focus. The value-related issues that arose were often unrelated to research ethics. Instead, they involved community matters such as the location of the PRC or the research topics to be pursued.

The concept of an equal partnership in community-based research between researchers and community members (reflected in values 3 and 9 and implied in other items) differs from the concept of a more paternalistic relationship between researchers and participants in traditional clinical research. The focus in traditional clinical research is on protecting the participant from the researcher, a concept that is not emphasized in the PRC's *Statement of Community Values.* Rather, the emphasis is on the belief that if properly informed and treated as an equal, the community can protect its own interests. One manifestation of this perspective is the committee that reviews each protocol. Although occasionally referred to as a *community IRB,* it differs from an IRB in that it is composed entirely of community representatives. The committee's ability to review projects is enhanced by the fact that protocols in health promotion research are generally less technical than protocols in traditional clinical research. In addition, in the past, the term "community laboratory" has been used in community-based research. We have discouraged its use because of the implication that community residents are analogous to subjects in a laboratory.

The PRC was not the first community-based research initiative at Morehouse School of Medicine, and the existence of a relatively robust program of community-based research provided the foundation on which to build the PRC. However, the PRC has provided a home base for community research, and it is now recognized as an important part of the school's research portfolio. In addition, community-based researchers often serve as mentors for public health students who are conducting their master's thesis research.

A partnership between community members and researchers is most successful if the partners can agree on a set of values to govern research. The set of community values developed by the Morehouse School of Medicine PRC Community Coalition Board reflect generally accepted ethical principles as well as community priorities. Attention to the *Statement of Values* has helped us build a stable relationship that benefits the community and the research enterprise.

## Figures and Tables

**Table 1:** Morehouse School of Medicine Prevention Research Center, *Research Priorities*

The Community Coalition Board of the Morehouse School of Medicine Prevention Research Center has established as a primary goal the promotion of holistic health among African Americans and other minority populations. The concept of holistic health reflects the World Health Organization definition of health: the total physical, mental, and social well-being of the individual and not merely the absence of disease or infirmity.
The Community Coalition Board is cognizant of the disparities in health status between the African American population and the white population in the United States, as reflected both in mortality rates and in other indicators of health status, such as years of potential life lost. These disparities indicate the extent to which the African American population has not reached its health potential. This is true of other minority populations as well, although to a lesser degree. The board is aware of the particularly disadvantaged status of African American males.
This background leads the board to establish the following as priorities for projects to be carried out by the Prevention Research Center:
Projects which, if successful, will contribute to a reduction in the disparity in health status between the white population and the African American population or other minority populations. Projects which, if successful, will contribute to improving the health status of African American males. Projects which, if successful, will reduce injustice, including environmental injustice.
Projects being considered by the Prevention Research Center should also be evaluated on the following criteria:
They should not violate community values or standards. They should have the potential to benefit the community through a health promotion intervention. Projects that propose simply to gather data should include in the proposal information on how the data-gathering process will lead to an intervention or otherwise improve the health of the community. Their effectiveness should be subject to evaluation and, if effectiveness can be demonstrated, they should be replicable in another setting.

**Table 2:** Morehouse School of Medicine Prevention Research Center, *Statement of Community Values*

Policies and programs should be based on mutual respect and justice for all people, free from any form of discrimination or bias. All people have a right to political, economic, cultural, and environmental self-determination. The community has the right to participate as an equal partner at every level of decision making, including needs assessment, planning, implementation, enforcement, and evaluation. Principles of individual and community informed consent should be strictly enforced. The community repudiates the targeting of people of color and lower socioeconomic status for the purpose of testing reproductive and medical procedures and vaccinations. Present and future generations should be provided an education that emphasizes social and environmental issues, based on our experience and an appreciation of our diverse cultural perspectives. Research processes and outcomes should benefit the community. Community members should be hired and trained whenever possible and appropriate, and the research should help build and enhance community assets. Community members should be part of the analysis and interpretation of data and should have input into how the results are distributed. This does not imply censorship of data or of publication, but rather the opportunity to make clear the community's views about the interpretation prior to final publication. Productive partnerships between researchers and community members should be encouraged to last beyond the life of the project. This will make it more likely that research findings will be incorporated into ongoing community programs and therefore provide the greatest possible benefit to the community from research. Community members should be empowered to initiate their own research projects that address needs they identify themselves.
